# Heterogeneous validity of daily data on symptoms of seasonal allergic rhinitis recorded by patients using the e‐diary AllergyMonitor®

**DOI:** 10.1002/clt2.12084

**Published:** 2021-12-15

**Authors:** Stephanie Dramburg, Serena Perna, Marco Di Fraia, Salvatore Tripodi, Stefania Arasi, Sveva Castelli, Danilo Villalta, Francesca Buzzulini, Ifigenia Sfika, Valeria Villella, Ekaterina Potapova, Maria Antonia Brighetti, Alessandro Travaglini, Pier Luigi Verardo, Simone Pelosi, Paolo Maria Matricardi

**Affiliations:** ^1^ Department of Pediatric Respiratory Medicine, Immunology and Critical Care Medicine Charité‐Universitätsmedizin Berlin Corporate Member of Freie Universität Berlin and Humboldt‐Universität zu Berlin Berlin Germany; ^2^ Pediatric Allergology Unit Sandro Pertini Hospital Rome Italy; ^3^ Allergology Service Policlinico Casilino Rome Italy; ^4^ Translational Research in Pediatric Specialities Area Division of Allergy Bambino Gesù Children's Hospital IRCCS Rome Italy; ^5^ Pediatric Allergology Unit Department of Pediatric Medicine Bambino Gesù Children´s Research Hospital (IRCCS) Rome Italy; ^6^ Department of Immunology‐Allergy “S.Maria degli Angeli” Hospital Pordenone Italy; ^7^ Department of Biology University of Rome “Tor Vergata” Rome Italy; ^8^ Center of Aerobiology ARPA Pordenone Italy; ^9^ TPS Production S.r.l. Rome Italy

**Keywords:** allergic rhinitis, mHealth, patient‐generated data, patient‐reported outcomes, symptom scores

## Abstract

**Background:**

Patient‐generated symptom and medication scores are essential for diagnostic and therapeutic decisions in seasonal allergic rhinitis (SAR). Previous studies have shown solid consistencies between different scores at population level in real‐life data and trials. For clinicians, the evaluation of individual data quality over time is essential to decide whether to rely on these data in clinical decision‐making.

**Objective:**

To analyze the consistency of different symptom (SS) and symptom medication scores (SMSs) at individual level in two study cohorts with different characteristics and explore individual patient trajectories over time.

**Methods:**

Within the pilot phase of the @IT.2020 project on diagnostic synergy of mobile health and molecular IgE assessment in patients with SAR, we analyzed data of 101 children and 93 adults with SAR and instructed them to record their symptoms and medication intake daily via the mobile app AllergyMonitor®. We then assessed the correlation between different SMS and a visual analogue scale (VAS) on the impact of allergy symptoms on daily life at population and individual level.

**Results:**

At population level, the Rhinoconjunctivitis total symptom score (RTSS) correlated better with VAS than the combined symptom and medication score (CSMS). At individual level, consistency among RTSS and VAS was highly heterogeneous and unrelated to disease severity or adherence to recording. Similar heterogeneity was observed for CSMS and VAS.

**Conclusions:**

The correlation of clinical information provided by different disease severity scores based on data collected via electronic diaries (e‐diaries), is sufficient at population level, but broadly heterogeneous for individual patients. Consistency of the recorded data must be examined for each patient before remotely collected information is used for clinical decision making.

## 1 INTRODUCTION

To date, allergen‐specific immunotherapy (AIT) is the only disease‐modifying treatment for seasonal allergic rhinitis (SAR),^1^ a non‐communicable disease affecting millions of citizens around the globe.^2^, ^3^ Although a clinical benefit and cost effectiveness have been proven in several settings,^4^, ^5^, ^6^, ^7^, ^8^ the treatment is long, expensive and demanding for patients who need to adhere to regular intakes or injections over several years. Therefore, international guidelines suggest, that AIT should only be prescribed for patients whose allergy symptoms are not sufficiently controllable with symptomatic pharmacological treatment and preventative measures, such as allergen avoidance.^9^

To enable an informed and shared clinical decision‐making, a standardized assessment of disease severity and control is usually performed *retrospectively* using validated questionnaires and criteria, such as the widely applied allergic rhinitis and its impact on asthma (ARIA) guidelines.^10^ Recently, several digital and mobile health technologies have been proposed to facilitate the *prospective real‐time* collection of patient‐generated data on symptom severity, medication intake or allergen exposure.^11^, ^12^, ^13^, ^14^, ^15^ Patients are being asked to enter their clinical symptoms mainly via user‐friendly electronic diaries (e‐diary) while exposure data are being collected through national or local pollen monitoring stations or networks. A merged report of the data then gives patients and attending healthcare professionals a comprehensive overview on the individual disease severity, patient compliance and symptom control.

The diagnostic usefulness of clinical e‐diaries, however, strongly depends on both, the patient's adherence to compilation and the quality of entered data. While the adherence to symptom recording is easily assessed by measuring the ratio of days with and without recordings during a fixed time period,^16^ data quality assessment is more complex and less intuitive.^17^ Recently, a large investigation evaluated *at population level* the validity, reliability and responsiveness of several visual analogue scales (VARs) for allergic rhinitis based on data collected via the mobile app MASK‐air®.^18^ The study demonstrated in a large data set, that the examined approach can be reliably used to monitor population subsets of allergic patients, for example, in trials investigating the efficacy of AIT.^19^

In addition to the use of patient‐generated data at population level, individual recordings may provide valuable insights, making data quality assessment for single patients essential. This assessment is particularly relevant for the appraisal of symptom severity within the daily practice of attending physicians deciding for or against the prescription or cessation of AIT.^20^ Before any data‐based decision making, the doctor should ascertain whether the amount (adherence)^16^ and quality (validity) of collected data is high enough.

A crucial question is, therefore, whether the level of consistency between the information on symptom severity (assessed by means of RTSS) and its impact on daily life (assessed via VAS) is uniform or heterogeneous for an individual patient. To answer this research question, we have analyzed the intra‐patient, day‐by‐day internal consistency of RTSS and VAS in two distinct cohorts of patients with SAR prospectively examined during the pollen season in the context of the @IT.2020 pilot project.

## 2 MATERIALS AND METHODS

### 2.1 Study population

The @IT.2020 project aims at developing and testing diagnostic algorithms integrating molecular allergology and digital health for the prescription of AIT in patients with SAR. In its pilot phase, 200 patients with a diagnosis of SAR were recruited before the pollen season in 2016, relevant for each of the patients, started. Children and adolescents (*n* = 101) were seen and recruited in the outpatient clinic of the Department of Pediatrics, “Sandro Pertini” Hospital, in Rome, while adult participants (*n* = 99) were recruited at the Department of Allergy, “S.Maria degli Angeli” Hospital, in Pordenone (North‐East Italy). Participants with complete data sets for both study visits as well as the symptom monitoring period were included in the present analysis (*n* = 101 for Rome; *n* = 93 for Pordenone). The inclusion criteria were (a) age 10–18 years (Rome), 18–60 years (Pordenone); (b) a history of SAR with/without asthma in one of the two last pollen seasons; (c) Italian speaking; (d) smartphone use/availability. The exclusion criteria were (a) previous pollen‐allergen AIT; (b) any other severe chronic disease, whose symptoms and/or therapy may mimic or mask the clinical picture of SAR (e.g., immune‐mediated disorders, immune deficiencies, chronic sinusitis or nasal polyposis); (c) living >20 km from/outside the climatic area served by the local aerobiological centre. Patients (or their parents in case of patients minor than 14 years of age) were interviewed and all patients underwent skin prick tests (SPT) as well as a blood drawing. All participants and/or their parents or tutors gave their informed written consent. The study design and procedures have been approved by the local ethical committees. Further details on the study population are published elsewhere.^21^

### 2.2 Study design

During the recruitment visit (T0), all patients filled a clinical questionnaire including selected sections from internationally validated questionnaires (International Study of Allergy and Asthma in Childhood (ISAAC)^22^; ARIA^23^ and Global Initiative for Asthma (GINA)^24^). A diagnosis of SAR was made based on the presence of (a) nasal and/or eye symptoms (apart from cold) for at least 3 weeks during one of the two last pollen seasons and (b) positive SPT (wheal reaction ≥3 mm) in accordance with clinical history and local pollination period. SPT were performed using a predefined panel of commercial extracts (ALK‐Abelló, Milan, Italy) of outdoor and indoor aeroallergens. Allergen‐specific IgE antibodies were assessed using a customized immunoblot test for pollen allergy diagnosis (Euroimmun, Lübeck, Germany).^25^ During the T0 visit, the Allergy.Monitor® app was downloaded, installed and tested on the patient's phone (or the phone of a legal guardian in the case of patients being minor 14 years of age and/or not owning a personal smartphone). Instructions for the correct recording of data as well as the timeframe of individual monitoring were given by the study nurse according to the individual monitoring periods previously established by the study doctor according to the expected pollination period(s) of clinically relevant pollen for every patient. Patients and/or their parents or tutors were asked to monitor symptoms and medication intake once per day during the potentially clinically relevant pollination periods. The grass peak pollen season (4 May to 28 June) was the only observation period relevant in all the patients recruited in Rome, while patients in Pordenone recorded their data during different periods arbitrarily prescribed by the local allergist and partially or totally overlapping with individual pollen seasons (*fagales*, pellitory, grass and others). After the individual monitoring period, all patients were invited to a final visit (T1) during which clinical questionnaires were repeated.

### 2.3 AllergyMonitor® app for data collection on symptom severity and medication intake

Allergy.Monitor® [Technology Project and Software (TPS) Production, Rome, Italy] is a mobile application designed for daily reporting of symptoms and medication intake related to SAR and/or asthma with a front‐end (i.e., patient app) and back‐office (i.e., doctor's website). In this study, all patients were asked to monitor their symptoms and medication intake via the AllergyMonitor® mobile app during an individually prescribed monitoring period according to the flowering periods of potentially eliciting allergen sources. The questionnaires included four questions on nasal symptoms (sneezing, rhinorrhea, nasal pruritus and nasal congestion), three on ocular symptoms (red eyes, itchy eyes and watery eyes), and three questions on the personal, scheduled medication intake (antihistaminic drugs, local steroids and systemic steroids). At the end of every data entry, users were asked to indicate the overall impact of their allergy symptoms via a continuous VAR (see below for detailed information). Patients could only submit a complete questionnaire to ensure the completeness of daily data sets. In the case of not entering any data for two consecutive days, users received an automatic alert message on their mobile phone or by email; after 3 days of missed reporting, the alert was followed by a phone call from the study physician or nurse. Further details on the @IT.2020 pilot study are available elsewhere.^21^

### 2.4 Used symptom (and medication) scores

For the present analysis, we calculated the following, widely used symptom (and medication) scores: (i) the Rhinoconjunctivitis total symptom score (RTSS; range: 0–18)^26^; (ii) the combined symptom and medication score (CSMS; range: 0–6)^27^; and iii) the VAR on general perception of allergy symptoms (VAS; range 0–10).^10^ While symptom severity assessment was performed using a four‐item self‐rating scale representing the severity levels (no symptoms, mild, moderate and severe) via four different emoticon faces, the general perception of allergy symptoms was assessed via the question ‘How do you feel in relation to your allergy symptoms today?’ and a continuous VAS for answer ranging from 0 (very good) to 10 (very bad). The interpretation of VAS does not require the application of a formula. RTSS and CSMS were automatically calculated within the Allergy.Monitor App using the answers on nasal (sneezing, rhinorrhea, nasal pruritus and nasal congestion) and ocular (itching, watery eyes) symptoms as well as medication intake (antihistaminic drugs, local steroids and systemic steroids). For details on the score calculation, please see Table e1.

### 2.5 Statistical analysis

Data were summarized as numbers (*n*) and frequencies (%) if they were categorical and as mean/median and standard deviation (SD)/interquartile range (IQR) if quantitative. The prevalence of atopic sensitization (SPT ≥ 3 mm) to airborne allergens was evaluated. For the pollen period considered, adherence values were calculated for each subject by assessing the number of filled reports within the individually prescribed monitoring period during the grass pollen season. For each trajectory of disease severity scores, five parameters were calculated considering the full set of data within the given observation period: (A) average value; (B) peak value; (C) cumulative value; (D) number of days above threshold; (E) fraction (%) of the days above threshold among the days with recording data To quantify the degree to which two scores were related, linear regression coefficient (beta) and coefficient of determination *R* ^2^ were calculated for RTSS and VAS at population level for all samples analysed and by centre, and at individual level considering RTSS as a dependent variable. Thresholds for low, medium, and high grades of correlation between RTSS and VAS were arbitrarily established at *R* ^2^ < 0.2, *R* ^2^ = 0.21–0.6 and *R* ^2^ < 0.6, respectively. A *p* < 0.05 was considered statistically significant. Statistical analyses were performed with R Core Team (2014), version 3.2.3.

## 3 RESULTS

### 3.1 Study population and pollen season

The present analysis includes 101 children (age 13.7 ± 2.8) and 93 adults (age 34.3 ± 14.4) meeting the previously mentioned inclusion criteria. Male gender was slightly more frequent among paediatric patients (62.4% vs. 55.9%) and the entire population is characterized by a predominantly persistent classification of allergic rhinitis symptoms as assessed by retrospective questionnaire during T0 according to ARIA criteria. Concerning severity, children and adolescents presented a heterogeneous clinical picture, while 90/93 (96.8%) of the adult patients classified as moderate to severe (Table 1). The most frequently observed allergic comorbidities among children and adolescents were oral allergy syndrome (32.2%), atopic dermatitis (28.3%) and allergic asthma (27.8%), while allergic asthma (25.8%), oral allergy syndrome (24.7%) and atopic dermatitis (11.8%) were the predominant comorbidities in the adult study population. In Rome, most participants were sensitized to grass pollen with 97% having a positive SPT to timothy grass and 90.1% reacting to Bermuda grass. In Pordenone, the prevalence of positive SPT to grass pollen was lower (80.6% with SPT positivity to Timothy grass and 44.1% to Bermuda grass), but still the most frequent sensitization (Table S1). In Rome, grass pollen concentrations in the air ranged from 0 to 199 grains/m^3^ and the local adaptation of season criteria of the European Academy of Allergy and Clinical Immunology (EAACI)^28^ resulted in a whole grass pollen season for 2016 from 13 April to 28 July, as well as a peak grass pollen season between 4 May and 28 June 2016. The median follow‐up period for recorded symptoms was 61 (Q1: 55; Q3: 88) days.

**TABLE 1:** Characteristics of the study population

	Rome (*n* = 101)		Pordenone (*n* = 93)	
	*n*	%	*n*	%
Male gender	63	62.4	52	55.9
Age (y) (mean, SD)	13.7	2.8	34.3	14.4
Allergic rhinitis				
Age at onset (y) (median, IQR)	6	(4–8)	15	(8–22)
ARIA classification at T0				
Mild Intermittent	19	18.8	1	1.1
Mild persistent	31	30.7	2	2.2
Mod./Severe Intermittent	11	10.9	17	18.3
Mod./Severe persistent	40	39.6	73	78.5
Other allergic comorbidities				
Allergic asthma	28	27.8	24	25.8
Oral allergic syndrome	32	32.3	23	24.7
Urticaria/Angioedema	19	19.2	8	8.6
Atopic dermatitis	28	28.3	11	11.8
Gastro‐intestinal disorders	4	4.0	1	1.1
Anaphylaxis episode	10	10.1	1	1.1
Other	5	5.1	2	2.2

### 3.2 Correlation of RTSS, VAS and CSMS at population level

**FIGURE 1:**
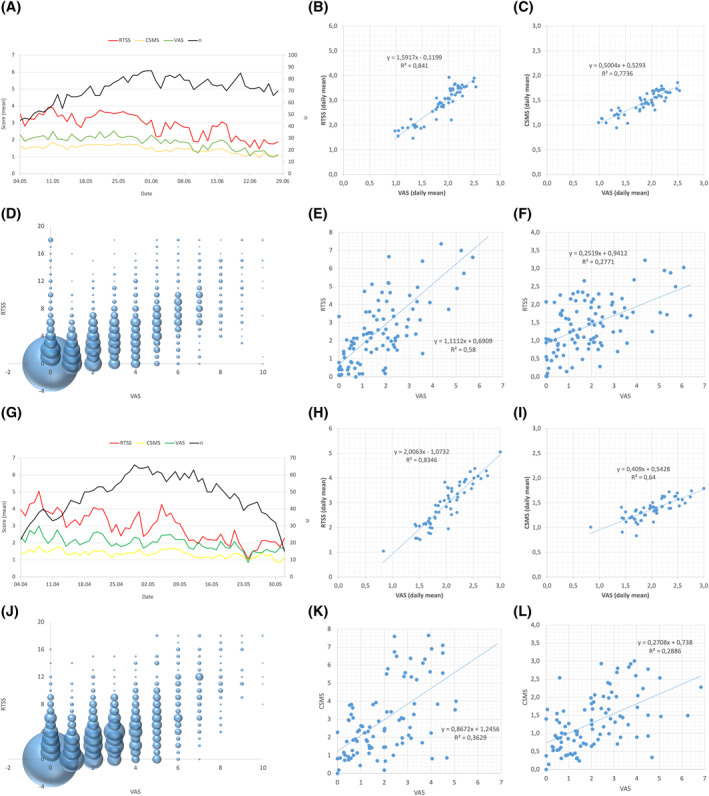
(i) Average population values of RTSS (0–18 points, red graph), CSMS (0–6 points, yellow graph), and VAS (0–10 points, green graph) over time in Rome (RM) (A) and Pordenone (PN) (G); (ii) correlation of daily mean values of RTSS and VAS for RM (B) and PN (H); (iii) correlation of daily mean values of CSMS and VAS for RM (C) and PN (I); (iv) consistency between RTSS and VAS at individual timepoints of recording (the bubble size indicates the number of data sets recorded with the respective combination of both scores) in RM (D) and PN (J); (v) the association of individual patient averages for RTSS and VAS in RM (E) and PN (K); and (vi) the association of individual patient averages for CSMS and VAS in RM (F) and PN (L)

During the reporting period, with a mean of 70.6 days (95% CI 64.9–74.4), the average adherence to symptom recording was 82.3% (SD 13.7) in Rome.^16^ In this centre, the trajectories of the three scores correlated well at population level over time during the grass pollen season (Figure 1A). The comparison of daily population averages of VAS explain the variation in RTSS with an *R* ^2^ of 0.841 (Figure 1B). Further, the analysis of individual data points of RTSS and VAS shows, that there is a broad range of combinations in terms of RTSS and VAS levels with the majority of data points (registered, individual daily questionnaires) ranging in the lower third of both scores (Figure 1D). A similar picture results from the comparison of individual RTSS and VAS averages at patient level (Figure 1E), where a lower correlation than at population level (*R* ^2^ = 0.58) can be observed. Regarding the correlation of CSMS and VAS, daily averages show a similar trend than for RTSS and VAS with values mainly in the lower third of both scores and an *R* ^2^ of 0.77 (Figure 1E). The individual averages of CSMS and VAS show a much lower correlation (*R* ^2^ = 0.28) and broader spreading (Figure 1F).

In Pordenone, the mean adherence to symptom recording was 85.7% (SD 13.9) during the reporting period which lasted on average 48.2 (95% CI 44.6–51.7) days.^16^ Also here, the trajectories of average RTSS, CSMS and VAS show a consistent trend over time (Figure 1G) and population averages of VAS explain well the RTSS variability with an *R* ^2^ of 0.834 (Figure 1H). In Pordenone, as in Rome, the correlation was lower for CSMS and VAS (*R* ^2^ = 0.64) (Figure 1I) and the majority of data points of RTSS and VAS ranged in the lower third of both scores (Figure 1J). Interestingly, the variability of individual average values of VAS and RTSS (Figure 1K), as well as VAS and CSMS (Figure 1L) is also high in Pordenone, reflecting the trends observed in Rome with correlations of *R* ^2^ = 0.36 and *R* ^2^ = 0.29, respectively.

### 3.3 Broad range of correlation between RTSS and VAS at individual level

**FIGURE 2:**
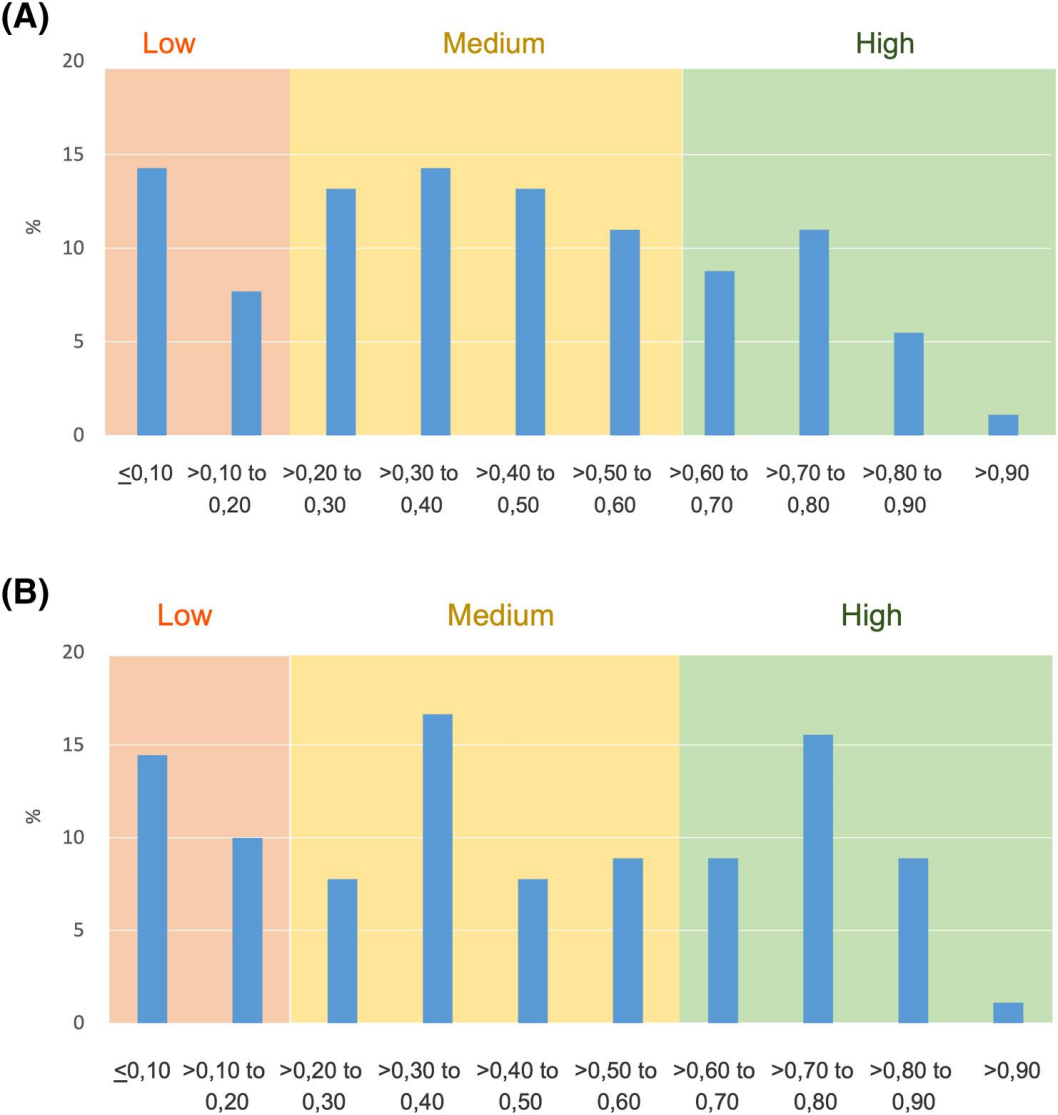
Frequency distribution of the coefficient of determination (*R* ^2^) (B, D) between RTSS score and VAS score in Rome (A) and Pordenone (B). Arbitrarily established cut‐offs are: *R* ^2^ 0–0.2 for low, 0.2 < *R* ^2^ ≥ 0.6 for medium, and 0.6 < *R* ^2^ for high correlation

The analysis of correlation between RTSS and VAS at individual level, performed through linear regression and expressed by the coefficient of determination (*R* ^2^) (Figure 2A and B) resulted in a heterogeneous picture within each centre but similar distributions between the study centres. A very wide range of patterns have been observed, from absence of consistency to an almost perfect or parallel day by day trend of the trajectories of daily RTSS and VAS values. Altogether, we have arbitrarily distinguished three subsets of patients with high, medium, and low or absent consistency. The frequency distribution of the patients in the three categories was similar in Rome (Figure 2A) and Pordenone (Figure 2C). In both centres, for each increasing unit of VAS, an average increase of about 1.5 units (1.42 in Rome, 1.63 in Pordenone) in the RTSS was registered. This trend is not surprising, considering that the VAS scale ranged from 0 to 10, while the scale of RTSS covers 0 to 18 points.

### 3.4 Individual patterns of RTSS and VAS trajectories

**FIGURE 3:**
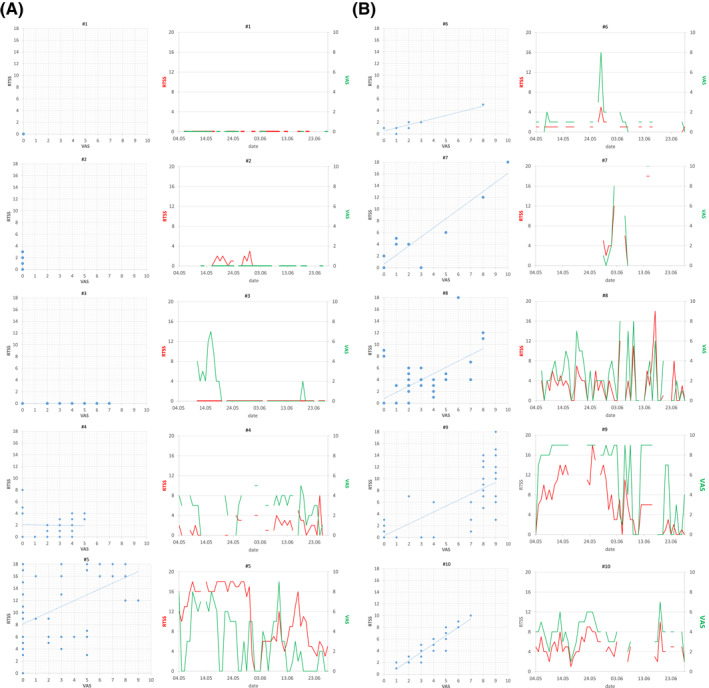
Individual patient slopes (#1–#10) of RTSS (red) and VAS (green) with their respective correlation (scattergrams) in Rome (A) and Pordenone (B). Panel a and b report patients with low (#1–#5) and high (#6–#10) level of consistency, respectively

The above reported heterogeneity of patterns is even clearer when the slopes of RTSS and VAS recorded by index patients are matched on the same timeline (Figure 3). Patients with a low correlation between the two scores (Figure 3A) showed the following trends: (#1) no symptoms or affection of daily life at all; (#2) low RTSS and no affection of daily life; (#3) variable impact of symptoms on daily life, but no symptoms indicated in RTSS; (#4) monitoring with large amounts of missing data and low overall consistency between RTSS and VAS; and (#5) comprehensive data set with medium to low consistency. Also, among patients with a high correlation between the two scores, different levels of data completeness could be observed (Figure 3B), ranging from very low (#6, #7), to medium (#10) and high (#8, #9). Nevertheless, independently from the amount of data available, almost perfect parallel trajectories could be observed in many patients (#6, #7, #9 and #10) but not in others, where the RTSS reached a maximum plateau (#8).

### 3.5 Lack of association between symptom (and medication) scores and diseases severity or adherence to recording

To answer the question whether a compliant and severely affected patient is also a patient with high quality compilation, we compared the level of consistency between RTSS and VAS scores to the level of adherence and the level of disease severity. We could not identify any association of the level of consistency between RTSS and VAS scores and the level of disease severity or adherence to compilation. This lack of association became clear in both clinical centres, in Rome (Figure E1a) and in Pordenone (Figure E1b).

## 4 DISCUSSION

Our results confirm that RTSS and VAS, two severity measures of allergic rhinitis, consistently correlate at population level. However, they also show that, at individual level, the level of consistency between RTSS and VAS is extremely variable ranging from strong associations to no consistency at all. The same trends could be observed for CSMS and VAS with generally weaker associations. The details and clinical implications of our observations deserve careful analysis and open new perspectives for further studies on the use of e‐diaries and their implementation in clinical decision making.

### 4.1 Score consistency at population level

Our observations have been made in two independent patient cohorts of the same pilot study. While the paediatric cohort in Rome consisted of 97% grass pollen allergic patients being followed throughout the local grass pollen season, the adult study population of Pordenone showed a more heterogeneous sensitization and recorded symptoms, as well as medication intake and affection of daily life independently from specific pollen seasons. In both cohorts, the parallelism in RTSS, CSMS and VAS trajectories and day‐by‐day correlation at population level was quite strong, even more pronounced than the intra‐patient average correlation. This echoes previous studies^18^ and confirms that external factors (e.g. specific pollen exposure in the paediatric cohort, Rome) contribute to converging information obtained through different questions on disease severity in populations with relatively homogeneous sensitization patterns (grass pollen sensitization in our case). Interestingly, the correlation of RTSS with VAS was higher than that of CSMS with VAS. This is in line with the idea that daily medication has no effect on VAS, as it is easy to use and has no side‐effects in patients with allergic rhinitis.

### 4.2 Heterogeneous consistency at individual level

Interestingly, we found that behind the good average score consistency measured at population level there is a broad heterogeneity of performance at individual patient level, which we found in both study cohorts. This demonstrates that patients are profoundly different in their capacity to provide similar answers to similar questions, repeated every day over a long monitoring period, even when they are participating in an observational study. However, most patients have good or excellent consistency, while patients with completely independent measures (RTSS and VAS) are a minority. Interestingly, the level of consistency is independent from adherence to recording. We observed patients with a relatively low adherence and high consistency of daily RTSS and VAS and patients with a very good adherence but low consistency between the two measures. This indicates that the quality of the data entered by the patient cannot be predicted on the basis of the quantity of the entered data that is, their adherence to compilation. Similarly, we also observed that the consistency in RTSS and VAS score was totally independent from the disease severity. Again, this contra‐intuitive outcome suggests that intention and attention in e‐diary compilation are two independent behavioural parameters.

### 4.3 Implications for the use of e‐diaries in trials or epidemiological studies

The observed parallel trajectories at population level highlight, that disease severity scores may be used interchangeably while studying populations or specific subsets, such as in trials or epidemiological studies. This is in line with previous observations, for example an analysis of electronically generated symptom data collected over 10 years with the Austrian and German Hayfever Diary.^29^ The authors analysed large data sets of pollen allergic patients over several pollen seasons and concluded that the exact method of symptom score calculation is not critical as all used computation methods showed similar trends over time. Therefore, the authors proposed a general symptom load index.^29^ Similarly, a comparison of six different disease severity daily average scores among a population of patients suffering from allergic rhinitis resulted in similar trajectories.^30^

While the importance of standardized outcome measures has been specifically underlined for clinical trials assessing the efficacy of AIT,^27^, ^31^ different patient‐reported outcomes are currently being evaluated by the ARIA expert group and an EAACI Task Force^31^ focusing on hypothesis versus data driven approaches in a very large data set collected via the mobile app MASK‐air®. Previous analyses of this remarkable dataset have shown good consistencies between different symptom data recorded via VARs and a variety of comparators such as quality of life (EQ‐5D), the Control of Allergic Rhinitis and Asthma Test (CARAT) score, and the Work Productivity and Activity Impairment Allergic Specific (WPAI‐AS).^18^, ^32^

### 4.4 Implications for the use of e‐diaries in routine allergy practice

In addition to these valuable previous outcomes, our observations of heterogeneity at individual level add important implications for the daily practice of the allergist. Our data suggest that, before taking clinical decisions for the individual patient based on data obtained through an e‐diary, the allergist must evaluate not only the completeness of the data (adherence),^16^ but also their quality and reliability. For example, a total lack of consistency between perceived severity of symptoms (RTSS) and perceived impact on the daily life (VAS) queries, whether the patient has rightly understood the target of the questions. Our results suggest that both parameters, *R* ^2^ and beta, may be used in an automatic report of the e‐diary to inform the doctor about the internal consistency of the information provided by the patient. Then, the doctor is alerted on the possibility of using the information given by the e‐diary or rather take decisions based on other diagnostic information. On the other hand, if the aim is using clinical information collected via an e‐diary, for example by automatically inserting data into a diagnostic algorithm or clinical decision support system (), then the measures of validity proposed here (*R* ^2^ and beta) should be used as a filter to limit the application of the CDSS only to cases with sufficiently consistent data. These hypotheses, originated by the present observations, deserve further studies and more articulated controls, possibly adopting external, independent measures of patient's understanding of this new diagnostic procedure and, more in general, of the patient's digital literacy.^33^, ^34^

## 5 STRENGTHS AND LIMITATIONS

An important strength of our study is the replication of similar results in two patient cohorts with different characteristics in terms of clinical centre, geographic region, pollen exposure, age and sensitization patterns. Important limitations include: (i) the data have been collected in the scope of an observational study and do not represent a real‐life scenario which should be the setting of future studies; (ii) data have been collected in only two different geographical regions, although Rome shares many characteristics of Mediterranean countries while Pordenone is more similar to central European regions. However, the results should be replicated in different geographical and cultural settings; (iii) as children and adults were recruited in geographically different centres, it is impossible to associate differences in the results with age or external factors, such as exposure to allergens or pollutants; and (iv) the presence of sensitizations to perennial allergens (e.g. mites, cat and dog) has not been taken into account in our analysis. Their role should be studied in depth within future studies.

## 6 CONCLUSIONS

The validity of clinical information provided by different disease severity scores based on data collected via e‐diaries, may be sufficient at population level, but is broadly heterogeneous at individual level. Data quality of the individual patient report must be examined before remotely collected information is used in clinical practice. Future studies in real‐life settings are required to further investigate risk factors of poor patient's performance and to elaborate strategies to improve not only patient's adherence, but also the quality of daily self‐reported clinical data.

## CONFLICT OF INTEREST

Paolo Matricardi reports grants and personal fees from TPS Software Production, outside the submitted work. Stephanie Dramburg reports fees from Bencard Allergie GmbH and Omron Healthcare Co. Ltd. Salvatore Tripodi and Simone Pelosi are co‐founder of TPS Software Production. Simone Pelosi reports personal fees from TPS Software Production. All other Authors declared no conflicts of interest.

## Supporting information

Supplementary Information S1
