# Supplementary material for: Heterogeneous validity of daily data on symptoms of seasonal allergic rhinitis recorded by patients using the e‐diary AllergyMonitor®
Source: Clin Transl Allergy. 2021 Dec 15;11(10):e12084. doi: 10.1002/clt2.12084 (PMC8674539; doi:10.1002/clt2.12084)

**Heterogeneous validity of patient-reported symptoms among patients with seasonal allergic rhinitis using the e-diary AllergyMonitor®**

Stephanie Dramburg^1^, Serena Perna^1^, M.Sc., Marco Di Fraia^1^, MD, Salvatore Tripodi^2,3^, MD, Stefania Arasi^1,4^, MD, PhD, Sveva Castelli^1^, Danilo Villalta^5^, MD, Francesca Buzzulini MD^5^, Ifigenia Sfika^2^, MD, Valeria Villella^2^, MD, Ekaterina Potapova^1^, Maria Antonia Brighetti ^6^, Alessandro Travaglini^6^, Pier Luigi Verardo^7^, Simone Pelosi, Eng ^8^ and Paolo Maria Matricardi^1*^, MD

**From the:**

^1^Department of Pediatric Respiratory Medicine, Immunology and Critical Care Medicine, Charité-Universitätsmedizin Berlin, Corporate Member of Freie Universität Berlin and Humboldt-Universität zu Berlin, 13353 Berlin, Germany

^2^ Pediatric Allergology Unit, Sandro Pertini Hospital, Rome, Italy

^3^ Allergology Service, Policlinico Casilino, Rome, Italy

^4^ Translational Research in Pediatric Specialities Area, Division of Allergy, Bambino Gesù Children's Hospital, IRCCS, Piazza Sant'Onofrio, 4, 00165 Rome, Italy

Pediatric Allergology Unit, Department of Pediatric Medicine, Bambino Gesù Children´s research Hospital (IRCCS), Rome, Italy.

^5^ Dept. of Immunology-Allergy, “S.Maria degli Angeli” Hospital, Pordenone, Italy

^6^ Dept. of Biology, University of Rome “Tor Vergata”, Rome, Italy

^7^ Center of Aerobiology, ARPA, Pordenone, Italy

^8^ TPS Production, Rome, Italy

**Table:** 2

**Figure:** 1

**^*^Corresponding author:**

Paolo M. Matricardi

Department of Pediatric Respiratory Medicine, Immunology and Critical Care Medicine

Charité Medical University

Augustenburgerplatz, 1

13353 Berlin, Germany

+49 30 450 566 406

+49 30 450 566 931

paolo.matricardi@charite.de

**Legend to the figure**

**Figure e1** – Correlation of RTSS (left column), CSMS (second column from left), VAS (second column from right) and adherence to recording (right column) with R2 in Rome (a) and Pordenone (b)

**Figure e1**


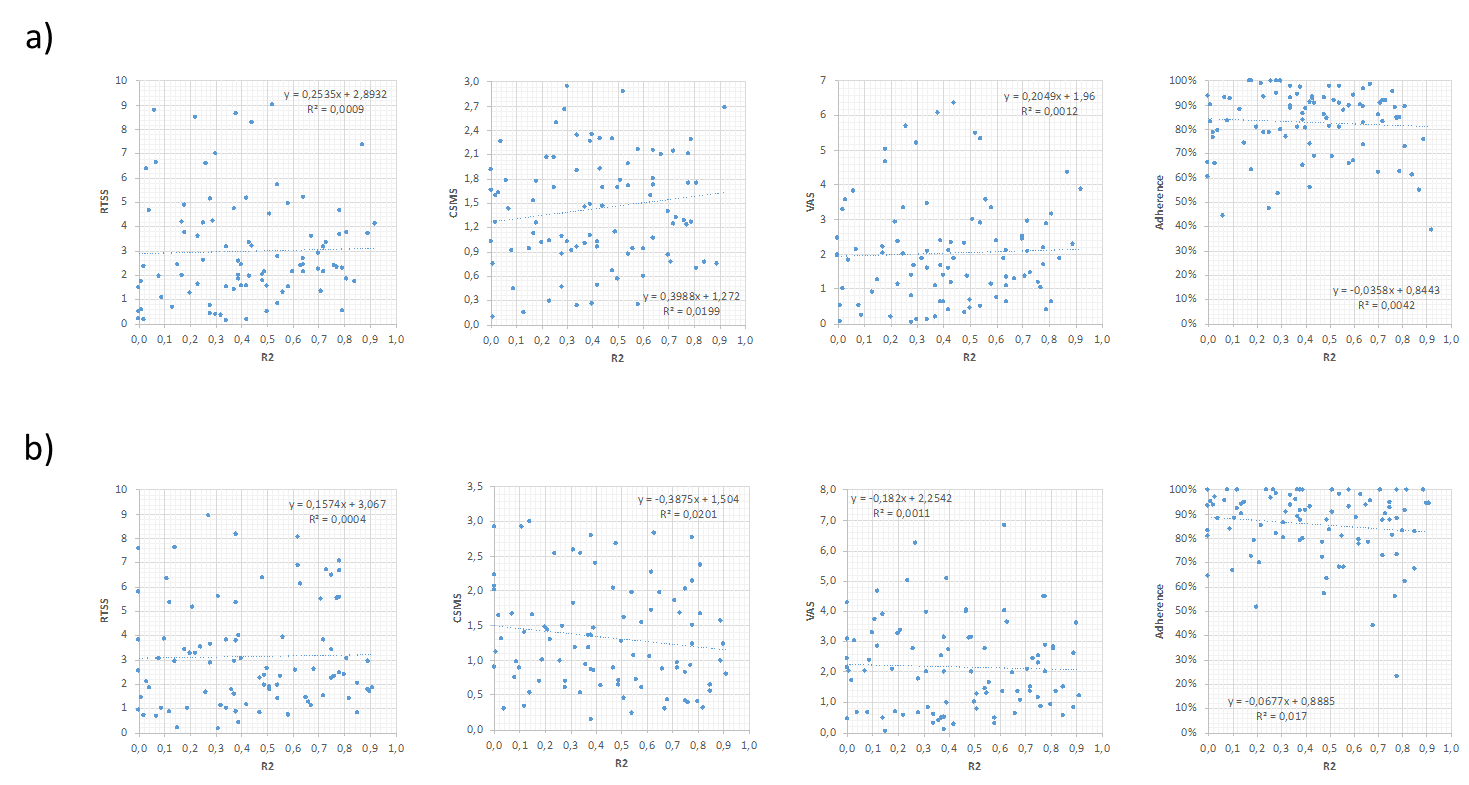

Supplement: Supplementary file 1 — Supplementary Information S1 [file CLT2-11-e12084-s001.docx]
